# Changes in radiocarpal and intercarpal arthrodesis incidence in Sweden: A 16-year retrospective analysis of 5 189 surgeries

**DOI:** 10.1371/journal.pone.0326885

**Published:** 2025-07-02

**Authors:** Michael Axenhus, Elsa Pihl, Cecilia Mellstrand Navarro, Viktor Schmidt

**Affiliations:** 1 Danderyd Wrist and Hand Initiative, Danderyd Hospital, Stockholm, Sweden; 2 Department of Orthopaedic Surgery, Danderyd Hospital, Stockholm, Sweden; 3 Department of Clinical Sciences at Danderyd Hospital, Karolinska Institutet, Stockholm, Sweden; 4 Department of Clinical Research and Education, Karolinska Institutet Södersjukhuset, Stockholm, Sweden; Upperline Health Inc., UNITED STATES OF AMERICA

## Abstract

Radiocarpal and intercarpal arthrodesis are common procedures for managing advanced arthritis, post-traumatic conditions, and failed interventions. This observational study analysed 5,189 cases of arthrodesis registered in the Swedish National Patient Register between 2008 and 2023 to identify surgical trends and future projections. Of these, 2,434 were radiocarpal and 2,755 intercarpal arthrodesis. Arthrodesis of the radiocarpal and intercarpal joints demonstrate fluctuating trends, regional disparities, and relatively equal rates between men and women. Younger men more often undergo intercarpal arthrodesis, while older women are in majority regarding radiocarpal arthrodesis, potentially reflecting differences in disease presentation, surgical indications, or occupational factors. A slight overall decline in arthrodesis rates suggests a shift towards alternative treatments like denervation or arthroplasty. These findings highlight the need for continued adaptation to evolving surgical techniques and standardised national care programs to optimize patient care and outcomes and mitigate inequalities in health care.

## Introduction

Arthrodesis of the radiocarpal and intercarpal joints are well-established surgical procedures designed to provide pain relief and restore stability [1–3]. The most frequent indications are advanced arthritis, post-traumatic conditions, or failed prior interventions [3–6].

Several techniques have been described to achieve solid fusion, with rigid dorsal plating being the standard for radiocarpal arthritis [1] and 2-, 3- or four-corner arthrodesis commonly used for intercarpal arthritis [7,8]. While arthrodesis effectively relieves pain, it necessitates adaptations in daily activities due to the loss of motion [1,3]. As an alternative, wrist arthroplasty has gained popularity among patients seeking pain relief while preserving mobility [3]. The evolution of hand prostheses implant designs, from early silicone implants to modern anatomic prostheses, has significantly improved outcomes, though challenges such as implant loosening and instability persist [4].

Previous research has primarily focused on different surgical techniques and clinical outcomes. Given the rising prevalence of wrist arthritis [9] and the continuous development of surgical techniques and implants, evaluating surgical trends is crucial. There is a lack of studies examining the trends of wrist arthrodesis. This study provides an analysis of these trends and future projections based on large-scale data from the Swedish National Patient Register.

## Materials and methods

### Study design and data source

This observational study using data derived from the Swedish National Patient Register (NPR), covering the period from 2008 to 2023. The NPR has been described as a validated source of data for epidemiological studies and have previously been used to study diseases and injuries in the wrist [10–12]. The methodology follows the principles outlined by the RECORD guidelines.

### Overview of Sweden’s healthcare system

Sweden provides universal healthcare to all residents, offering free access to emergency care, inpatient treatments, and outpatient services. There exists both private and public hospitals offering a variety of orthopaedic services. Each Swedish citizen is assigned a unique personal identification number (PIN) that remains unchanged in all contact with authorities and healthcare throughout life, which enables tracking of healthcare services across different facilities. This PIN is integrated into national healthcare databases, allowing for precise and comprehensive data aggregation.

### Data collection and registry information

The NPR serves as a robust database documenting inpatient medical care since 1964 and specialized outpatient care starting in 2001. Initially updated on an annual basis, the registry transitioned to monthly updates in 2021 to improve accuracy by including late-submitted and corrected records. NPR entries provide granular details, such as patient demographics, surgical procedures, and geographic distribution. Reporting to the NPR is compulsory for all healthcare providers and includes ICD-10 coded diagnoses and procedures categorized using the NOMESCO classification system [13,14].

### Criteria for participant inclusion

The study population consisted of individuals aged 15 and older who had been registered as having undergone a primary arthrodesis of the radiocarpal or intercarpal joints, recorded under the NOMESCO codes NDG40–41. Only patients with a valid Swedish personal identification number were considered eligible.

#### Inclusion criteria.

1. Residents of Sweden during the observation period (January 1, 2008, to December 31, 2023).
2. Individuals who had undergone a primary hand arthrodesis.

#### Exclusion criteria.

1. Patients below the age of 15.

### Statistical analysis

The dataset was segmented by sex, joint, age category, and region to analyse time-based trends. Data was extracted per 100 000 inhabitants and total number of surgeries. Differences in incidence rates across sexes for specific age brackets were tested using Student’s t-tests. To predict future trends, regression analysis was employed, and we fitted exponential, linear, logarithmic, polynomial, and power regression models for each incidence trend. Predictive analysis was based on the best-fitting model, with 95% confidence intervals (CI) applied where relevant. The year was used as the independent variable and incidence of fracture surgery was as the exposure. R^2^ value was used to determine best fit. The significance level was set at p < 0.05, with additional levels indicated as follows: * p < 0.05, ** p < 0.01, *** p < 0.001, **** p < 0.0001.

### Ethics

Since the research solely relied on publicly available data, it was exempt from requiring ethical approval according to Swedish law.

## Results

**Fig 1:**
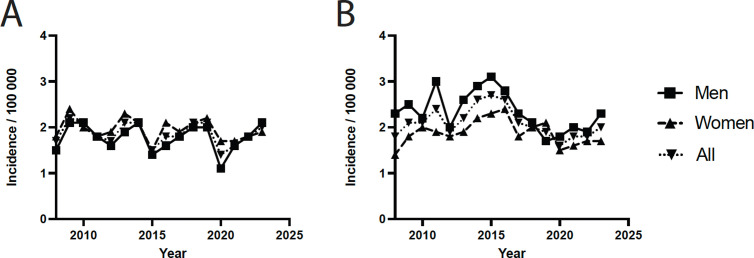
Trends of arthrodesis in the hand in Sweden during 2008 to 2023. A) Radiocarpal joint, B) Intercarpal joint.

A total of 5 189 patients, of whom 2434 registered with a radiocarpal and 2755 with an intercarpal arthrodesis, were identified during the study period (Table 1). In the case of the radiocarpal joint (Fig 1A), the incidence displayed noticeable fluctuations but no clear upward or downward trend over the years. Arthrodesis of intercarpal joints (Fig 1B) showed relatively stable incidence rates, with both sexes demonstrating similar trends and no substantial sex-based differences.

**Table 1:** Demographics and number of procedures performed for each patient and age group during the study period.

Procedure	Sex	Age group								Total
		15-24	25-34	35-44	45-54	55-64	65-74	75-84	85+	
NGD40	Women	49	57	124	191	296	235	100	10	1062
NGD40	Men	77	77	76	170	277	171	60	6	914
NGD41	Women	32	53	88	182	264	138	42	3	802
NGD41	Men	42	100	137	241	347	159	35	4	1065

**Fig 2:**
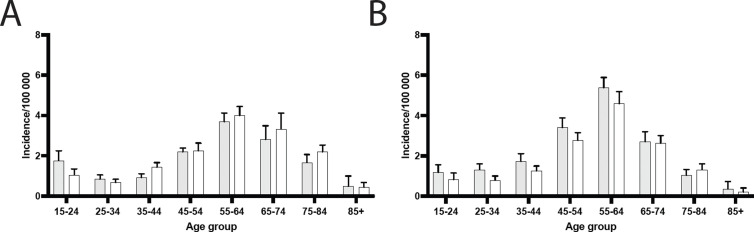
Age group differences in arthrodesis in the hand in Sweden during 2008 to 2023. A) Radiocarpal joint, B) Intercarpal joint. White indicates women and grey indicate men. Error bars indicate 95% CI.

Overall, the incidence increased with age, peaking in the 65–74 and 75–84 age groups before declining in the 85 + category (Fig 2). The radiocarpal joint demonstrated a gradual increase in incidence with age, with a more pronounced difference between sexes in older populations (Fig 2A). In the intercarpal joints the incidence peaked in the 75–84 group and declined thereafter, with men generally exhibiting higher rates across most age groups (Fig 2B).

Regional analysis of arthrodesis surgeries in Sweden from 2008 to 2023 demonstrated notable variations in the distribution of radiocarpal joints (A) and intercarpal joints (B).

**Fig 3:**
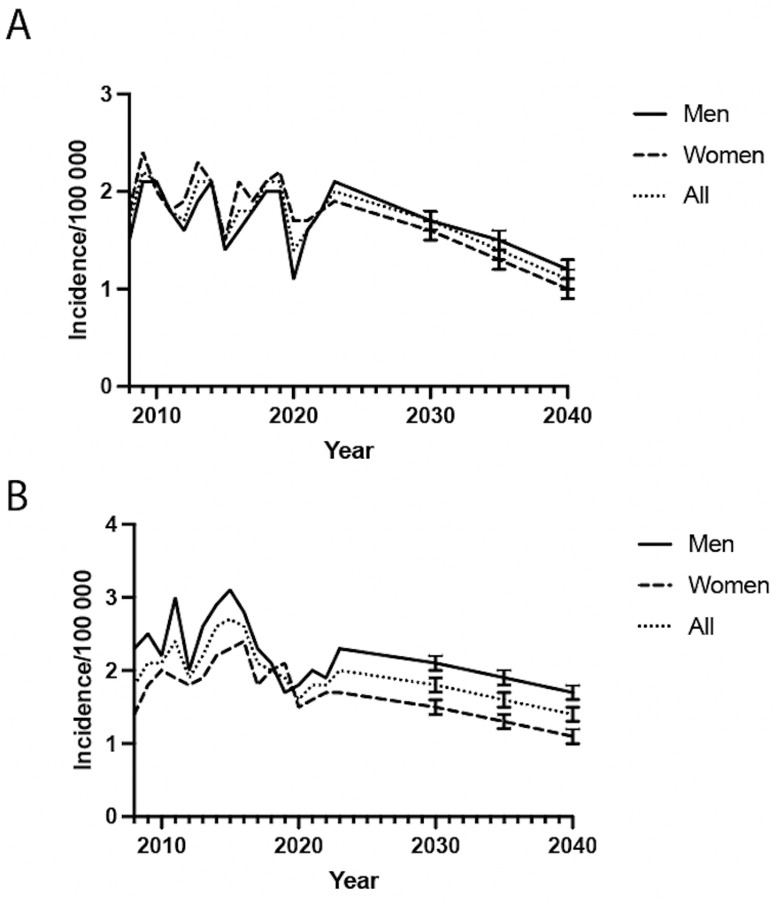
Predicted trends in the incidence of wrist hand arthrodesis procedures in Sweden from 2010 to 2040 for radiocarpal joint (A) and intercarpal joint (B).

Overall, the incidence of radiocarpal and intercarpal arthrodesis is expected to decrease over time, with the incidence showing a steady decline from approximately 2.0 per 100,000 in 2010 to below 1.2 per 100,000 by 2040 for radiocarpal joint arthrodesis (Fig 3A). Intercarpal joint arthrodesis is expected to decrease to approximately 1.5 by 2040 (Fig 3B).

## Discussion

The main findings of this 16-year national analysis of radiocarpal and intercarpal arthrodesis include fluctuating surgical trends, notable regional disparities, and relatively equal incidence rates between men and women.

**Fig 4:**
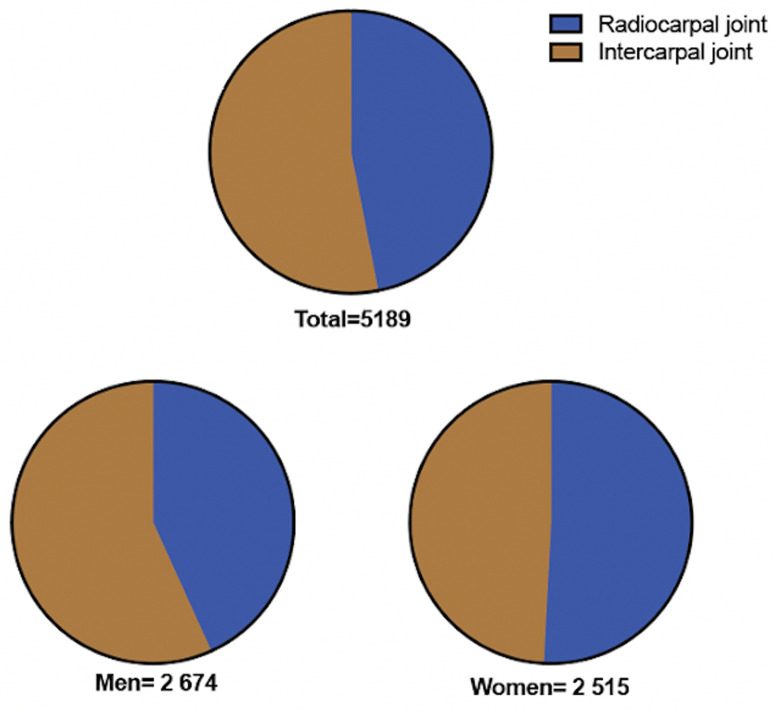
Distribution of wrist arthrodesis surgeries categorized by joint type and sex, during the period 2008–2023.

The two procedures are about as common (Fig 4), with women having a slightly higher incidence of radiocarpal arthrodesis and men more intercarpal arthrodesis (Fig 1). Since intercarpal arthrodesis is less limiting regarding motion, it is often used as a first step in treating localised wrist arthritis [3]. This might explain why it is being used more in slightly younger patients. It may be surprising that men undergo arthrodesis more frequently than women, given that existing literature reports a higher prevalence of joint disease in women. This is often attributed to factors such as the greater incidence of rheumatic diseases, hormonal influences on bone and cartilage metabolism, and women’s longer life expectancy [15–17]. Nevertheless, older women might be subjects to more arthroplasty in the wrist and hand as heavy manual labour is often considered a relative contraindication to arthroplasty [1,18]. Moreover, men might suffer from more post-traumatic arthritis in the wrist [19]. Large national studies on arthrodesis of the wrist and hand are scarce, however, in an analysis of the incidences in the US, Kopriva et al. found that four-corner arthrodesis and total wrist arthrodesis remained stable over time, with an increase of proximal row carpectomy [20].

For the radiocarpal joint, there were noticeable fluctuations with a slight downward trend over time (Fig 1A). Arthrodesis of intercarpal joints (Fig 1B) showed similar but more stable incidence rates. Both demonstrated significant dips in 2020 which can be explained by COVID-19. Regression modelling suggests an overall decline in arthrodesis surgery rates. The reason for overall slight downward trends in radiocarpal and intercarpal arthrodesis might indicate new treatment regimens prioritising denervation, excision procedures or arthroplasty before considering arthrodesis [1,3]. Better treatments for rheumatic diseases might also be a negative driver [21]. Future research could investigate whether these trends are influenced by variations in surgical indications or patient preferences, and whether they lead to improved postoperative outcomes.

**Fig 5:**
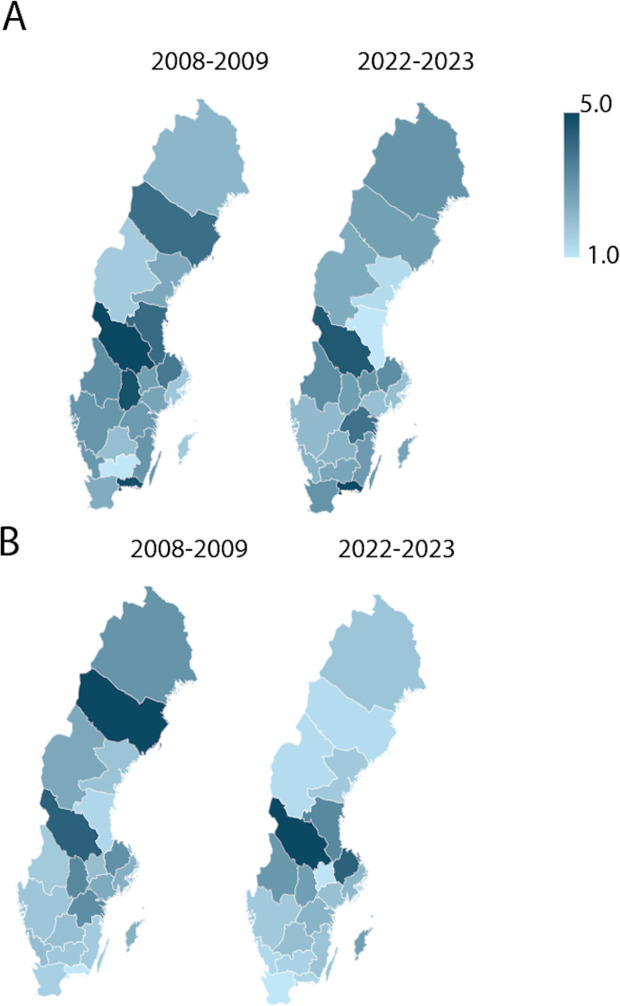
Regional analysis of arthrodesis surgeries in Sweden, categorized by joint type showing the average incidence during 2008-2009 compared to average incidence during 2022-2023. The surgeries are presented as radiocarpal joints (A), intercarpal joints (B).

Regional analysis identified substantial variation, with region Dalarna standing as having the highest incidences of both radiocarpal and intercarpal arthrodesis (Fig 5). Dalarna exhibit incidence rates up to 5 times of those of other regions, which was stable over time. The regional variations are consistent with previous national-level data on other musculoskeletal conditions and highlight the need for national guidelines to avoid unwarranted treatment variations [12,22,23]. The role of socioeconomic factors, healthcare infrastructure, and patient demographics in driving these regional imparities is yet to be explored.

Using registry data has inherent limitations and potential biases, including underreporting and miscoding of procedures. Furthermore, the study lacks clinical outcome data and patient-reported outcome measures, which are essential for assessing the impact of surgical interventions. While significant disparities were identified, the study does not investigate the underlying factors such as socioeconomic status, comorbidities, or other reasons for variations in regional healthcare practises. Lastly, although predictive modelling indicates declining rates of arthrodesis, future research is needed to validate these projections and examine the factors contributing to this trend.

## Conclusion

Arthrodesis of the radiocarpal and intercarpal joints demonstrate fluctuating trends with a decline over time, regional disparities, and relatively equal rates between men and women. Younger men more often undergo intercarpal arthrodesis, while older women are more frequently treated with radiocarpal arthrodesis, potentially reflecting differences in disease presentation, surgical indications, or occupational factors. A slight overall decline in arthrodesis rates suggests a shift towards alternative treatments like denervation or arthroplasty. These findings highlight the need for continued adaptation to evolving surgical techniques and standardised national care programs to optimize patient care and outcomes.
